# Impact of COVID-19 on Mitochondrial-Based Immunity in Aging and Age-Related Diseases

**DOI:** 10.3389/fnagi.2020.614650

**Published:** 2021-01-12

**Authors:** Riya Ganji, P. Hemachandra Reddy

**Affiliations:** ^1^Department of Internal Medicine, Texas Tech University Health Sciences Center, Lubbock, TX, United States; ^2^Departments of Neuroscience and Pharmacology, Texas Tech University Health Sciences Center, Lubbock, TX, United States; ^3^Department of Neurology, School of Medicine, Texas Tech University Health Sciences Center, Lubbock, TX, United States; ^4^Public Health Department of Graduate School of Biomedical Sciences, Texas Tech University Health Sciences Center, Lubbock, TX, United States; ^5^Department of Speech, Language and Hearing Sciences, School Health Professions, Texas Tech University Health Sciences Center, Lubbock, TX, United States

**Keywords:** COVID-19, SARS-CoV-2, Alzheimer’s disease, diabetes, obesity, immune response, mitochondrial dynamics, lifestyle

## Abstract

The coronavirus disease 2019 (COVID-19) has become a deadly pandemic with surging mortality rates and no cure. COVID-19 is caused by the severe acute respiratory syndrome corona virus 2 (SARS-CoV-2) with a range of clinical symptoms, including cough, fever, chills, headache, shortness of breath, difficulty breathing, muscle pain, and a loss of smell or taste. Aged individuals with compromised immunity are highly susceptible to COVID-19 and the likelihood of mortality increases with age and the presence of comorbidities such as hypertension, diabetes mellitus, cardiovascular disease, or chronic obstructive pulmonary disease. Emerging evidence suggests that COVID-19 highjacks mitochondria of immune cells, replicates within mitochondrial structures, and impairs mitochondrial dynamics leading to cell death. Mitochondria are the powerhouses of the cell and are largely involved in maintaining cell immunity, homeostasis, and cell survival/death. Increasing evidence suggests that mitochondria from COVID-19 infected cells are highly vulnerable, and vulnerability increases with age. The purpose of our article is to summarize the role of various age-related comorbidities such as diabetes, obesity, and neurological diseases in increasing mortality rates amongst the elderly with COVID-19. Our article also highlights the interaction between coronavirus and mitochondrial dynamics in immune cells. We also highlight the current treatments, lifestyles, and safety measures that can help protect against COVID-19. Further research is urgently needed to understand the molecular mechanisms between the mitochondrial virus and disease progression in COVID-19 patients.

## Introduction

**Figure 1:**
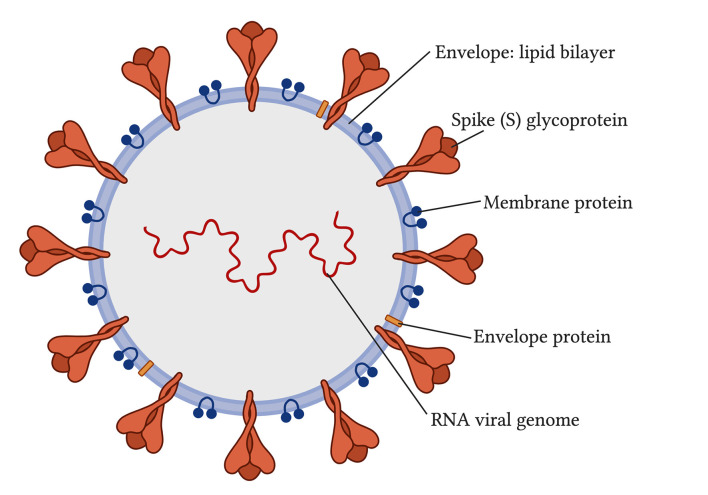
Structure of severe acute respiratory syndrome coronavirus type-2 (SARS-CoV-2; Vallamkondu et al., 2020).

Coronaviruses are viruses that come from the *coronaviridae* family and *Nidovirales* order (Vallamkondu et al., 2020). When viewed with electron microscopy, coronaviruses have a crown-like appearance caused by the spike glycoproteins on their envelopes (Figure 1; Vallamkondu et al., 2020). Coronaviruses can use human lung alveolar epithelial cells as host cells for their survival and replication. In 2019, a novel coronavirus emerged, referred to as Severe Acute Respiratory Syndrome coronavirus type-2 (SARS-CoV-2). It caused a worldwide pandemic with its disease, named by the World Health Organization (WHO) as the novel coronavirus disease discovered in 2019, or COVID-19.

COVID-19 causes a range of respiratory symptoms, varying with patient demographic makeup and medical history. These symptoms include sore throat, cough, fever, chills, headache, shortness of breath, difficulty breathing, muscle pain, and a loss of smell or taste (Huang et al., 2020; Li et al., 2020). It is transmitted *via* respiratory droplets and has a reproductive quotient (R0) of 2.2, meaning that a person infected with SARS-CoV-2 infects roughly 2.2 new individuals (Kandimalla et al., 2020). As of December 2020, there have been nearly 70 million cases worldwide and upwards of 1.5 million deaths [World Health Organization (WHO), 2020], and while many medications are being tested for efficacy, none have proven to be a one-for-all solution yet (Bhatti et al., 2020). Patients that are more likely to present with symptoms of COVID-19 are older individuals; the likelihood increases with age and with the presence of comorbidities such as hypertension, diabetes mellitus, cardiovascular disease, or chronic obstructive pulmonary disease (Yang et al., 2020; Zhang et al., 2020), as well as obesity or dementia (Holder and Reddy, 2020). Those with the highest mortality rate seem to be older, male patients with various comorbidities (Onder et al., 2020).

Given the prevalence of older individuals among those who acquired COVID-19, it is necessary to explore the mechanisms that could encourage a link between aging and COVID-19 so that treatment formation may be better directed, and health professionals can better assess risks in these patients. As humans age, the speed and strength of their immune response weaken due to the loss of certain immune tissues such as the thymus, as well as poorer energy metabolism at the cellular level with mitochondria. The energy in the cell comes in the form of adenosine triphosphate (ATP) and is made by mitochondria when the cell is fueled with oxygen, as well as by glycolysis in the absence of oxygen. Mitochondria are also known to interact with viral particles when they infect human host cells, engaging interferon and cytokine release, stimulating inflammation, and influencing viral survival and replication (Khan et al., 2015; Tiku et al., 2020). Studying mitochondrial-based immunity against the SARS-CoV-2 may give insight into why older individuals, with lessened mitochondrial efficiency, maybe worse equipped to face COVID-19.

The purpose of our study is to explore the molecular link between mitochondria in aged individuals and SARS-CoV-2. Furthermore, we also highlighted the role of various age-related comorbidities such as diabetes, obesity, and neurological illnesses in increased mortality rates amongst the elderly with COVID-19. We also explore current treatments, lifestyles, and safety measures that can help protect against COVID-19.

## Mitochondria and Immunity

Mitochondria are organelles with a double membrane that serves as a cell’s primary source of energy production in the form of ATP and contribute to homeostasis, cell proliferation, cell death, and synthesis of amino acids, lipids, and nucleotides. In the event of an infection, mitochondria contribute to immunity by engaging the interferon system, altering their structure, and inducing programmed cell death (apoptosis; Ohta and Nishiyama, 2011).

### Interferon Signaling and Mitochondria

Upon viral infection, the host’s innate immune system recognizes certain patterns, such as viral nucleic acid sequences or viral proteins, when they attach to receptors on host cellular membranes, intracellularly and extracellularly. Their recognition activates signaling pathways that lead to the inflammatory response. There are different types of receptors that these viral components attach to, including toll-like receptors and retinoic acid-inducible gene-I-like receptors (RLRs; Takeuchi and Akira, 2009). Toll-like receptors are involved in activating type-I interferon, an inflammatory cytokine, and chemokine production and are found on the cell surface, endosome, and endoplasmic reticulum membranes (Takeuchi and Akira, 2009). RLRs are the cytosolic receptors that start the production of type-I interferon in nonimmune cells (Takeuchi and Akira, 2009) and can be found on mitochondria (Tal and Iwasaki, 2009).

These RLRs detect viral RNA in the cytoplasm and are involved in the recognition of RNA viruses such as paramyxoviruses, Japanese encephalitis virus, influenza virus, and picornaviruses (Kato et al., 2006). However, certain subtypes of RLRs may be involved in the detection of certain DNA viruses as well. For example, adenovirus and Herpes Simplex Virus Type 1 have a DNA-dependent RNA polymerase III that affects RLRs and stimulates interferon-β production, and the Epstein-Barr virus produces small RNA fragments that can activate RLRs (Samanta et al., 2006; Cheng et al., 2007; Chiu et al., 2009).

A component of the signaling pathway stemming from RLR activation is a molecule known as mitochondrial antiviral signaling protein (MAVS; Kawai et al., 2005). MAVS is located on the outer mitochondrial membrane (OMM; Seth et al., 2005) and upon activation, triggers transcription factors that will result in additional interferon production (Zhang et al., 2014). In addition to MAVS, the mitochondria-associated protein called “stimulator of interferon genes” and the mitochondrial protein mitofusin 2 are also involved in RLR cascades or work with MAVS (Ishikawa and Barber, 2008; Yasukawa et al., 2009). This evidence goes to show that mitochondria are an important part of interferon signaling in the immune system. Some viruses alter MAVS levels to prevent interferon production; the influenza A virus (Varga et al., 2012), the measles virus (Xia et al., 2014), the Newcastle disease virus (Meng et al., 2014), and the Hepatitis C virus (Ohta and Nishiyama, 2011) reduce or degrade MAVS as a way to prolong survival by reducing interferon signaling.

### Mitochondrial Fission and Fusion Modulation

Viruses can manipulate mitochondrial fission and fusion to benefit viral survival (Holder and Reddy, 2020). Mitochondria can alter their structure through fission and fusion of their OMM and inner mitochondrial membrane (IMM), by functions involving GTPases related to dynamin (Tiku et al., 2020). The fusion of the OMM is mediated by proteins Mitofusin 1 and Mitofusin 2 *via* GTP hydrolysis, and fusion of the IMM is mediated by the protein optic atrophy 1, which is a GTPase present in the IMM (Tiku et al., 2020). Mitochondrial fusion is needed for the exchange of mitochondrial DNA, proteins, and metabolites (Archer, 2013). On the other hand, fission of the OMM is mediated by the cytosolic GTPase dynamin-related protein 1 (Drp1) *via* GTP hydrolysis (Mears et al., 2011). Upon finding a mitochondrial scission site, Drp1 interacts with mitochondrial fission factor and mitochondrial dynamics proteins 49 and 51 to constrict and cut the OMM (Mears et al., 2011; Loson et al., 2013). The mechanisms of mitochondrial fission are not well understood. Fission is needed for removing damaged parts of mitochondria to be cleared by mitophagy (autophagy of the mitochondria) and is needed during cell cycle replication (Mao and Klionsky, 2013). Thus, enhanced fission usually leads to increased mitophagy.

Some viruses may promote mitochondrial fusion to reduce the interferon pro-inflammatory response against viruses through a mechanism that involves mitofusin 2 inhibition of MAVS. For example, the dengue virus stimulates mitochondrial fusion *via* its nonstructural protein NS4B (Barbier et al., 2017), and HIV enhances fusion *via* its envelope protein gp120 (Fields et al., 2016). The SARS coronavirus (SARS-CoV-1) enhances fusion *via* its virulence factor ORF-9b (Shi et al., 2014). These virulence factors reduce the levels of Drp1, the fission-inducing protein, thus leading to unbalanced mitochondrial fusion, which is driven by mitofusin 2. As mitofusin 2 interacts with and inhibits MAVS, which typically increases interferons (Yasukawa et al., 2009) this can hinder the interferon response. Interestingly, SARS-CoV-1 uses the same ORF-9b to also reduce levels of MAVS directly, which further lowers the interferon response (Shi et al., 2014).

Some viruses induce mitochondrial fission to enhance mitophagy and alter the rate of apoptosis, usually *via* up-regulation or activation of Drp1 and/or degradation or inhibition of MAVS (Khan et al., 2015). Among these is the Hepatitis C virus *via* its core proteins and proteins E1-E2 (Kim et al., 2014), the Human cytomegalovirus *via* viral protein vMIA (McCormick et al., 2003), and the Hepatitis B virus *via* viral protein HBx (Kim et al., 2013). Fission mediated by these three particular viruses leads to inhibition of apoptosis so that the virus may survive for longer and further replicate (McCormick et al., 2003; Kim et al., 2013, 2014).

Due to the modulation by viruses on mitochondrial fusion and fission, their presence may lead to altered energy levels by way of mitochondrial count and form. Viruses that cause mitochondrial fission and lead to inhibition of apoptosis can allow viral particles to survive unharmed for longer. Many patients often feel weak from a lack of energy when infected with a viral illness. This may be due to the poorer mitochondrial energy production as a result of the increased fission.

### Cell Death

Apoptosis, or programmed cell death, is another important function of the cell influenced by the mitochondria. There is an extrinsic pathway to activate apoptosis, controlled by certain ligands binding to “death” receptors, and an intrinsic pathway that is controlled by mitochondria (Brenner and Mak, 2009). In this intrinsic pathway, the mitochondrial membrane is permeated and the mitochondrial membrane potential (MMP) is disrupted as the intermembrane space proteins spill into the cytoplasm (Shawgo et al., 2008). These proteins include cytochrome *c* (Liu et al., 1996), caspase-9 (Du et al., 2000), and apoptosis protease activating factor 1 that work together to form an apoptosome, which stimulates the final caspases to carry out cell death procedure (Cain et al., 2002).

MMP destabilization, which leads to apoptosis, is thought to occur by various mechanisms. First, there is the mechanism of selective OMM permeabilization (Kuwana et al., 2002). Bax and Bak, proteins that make up the Bax proapoptotic subfamily of Bcl-2 proteins, serve as pores on the mitochondrial membrane to maintain the MMP as well as release cytochrome c and calcium from within the mitochondria (Nutt et al., 2002). When another proapoptotic subfamily known as the BH3-only subfamily attaches to activated Bax/Bak, they enhance permeability and increase the chance of apoptosis (Chen et al., 2005). On the other hand, the antiapoptotic Bcl-2 subfamily (including Bcl-2 and Bcl-xL) can attach to activated Bax/Bak proteins and inhibit Bax/Bak by forming an antiapoptotic complex and leading to decreased apoptosis (Shawgo et al., 2008). A second mechanism involves the lipid bilayer makeup of the mitochondrial membrane and involves Bax creating rapid reorganization of the lipids that leads to structural stress and hole formation (Terrones et al., 2004). The last mechanism involves the stimulation of the permeability transition pore complex (PTPC) in the IMM (Shimizu et al., 1999). This is triggered by an overabundance of calcium or reactive oxygen species (Deniaud et al., 2008) and can be influenced by proteins from all parts of the mitochondria.

Viruses can use viral proteins that mimic Bcl-2 family members and other factors that are involved in the apoptosis pathways to manipulate the cell’s lifespan as they see fit. Table 1 demonstrates some common examples to illustrate this point.

**Table 1:** Viral Effects on apoptosis *via* the internal mechanism of mitochondrial influence.

ANTI-APOPTOTIC		
Virus; protein	Mechanism	Notes
Adenovirus; E1B-19K	Bcl-2 mimic	Han et al. (1996)
African swine fever virus; A179L	Bcl-2 mimic	Brun et al. (1996)
Myxoma virus; M11L	Bcl-2 mimic	Douglas et al. (2007)
Epstein-Barr virus; BHRF1	Bcl-2 mimic	Hickish et al. (1994)
Human herpesvirus 8; Kaposi’s sarcoma-associated Bcl-2	Bcl-2 mimic	Cheng et al. (1997)
Cytomegalovirus; vMIA	PTPC inhibition	Goldmacher (2002)
SARS-CoV-1; NSP15	Inhibited MAVS-induced apoptosis	Dose-dependent and with specificity as it did not inhibit staurosporine-induced apoptosis Lei et al. (2009).
**PRO-APOPTOTIC**		
**Virus; protein**	**Mechanism**	**Notes**
Human papillomavirus; E1 and E4	Disrupt the intracellular keratin network and cause mitochondria to accumulate next to the nucleus and lose MMP	Doorbar et al. (1991)
Vesicular stomatitis virus; matrix protein M	Interfere with the Bcl-2 family	Gadaleta et al. (2005)
Avian encephalomyelitis virus; 2C	Activates caspase-9	Liu et al. (2004)
Hepatitis C virus; NS4A	Disrupts MMP	Nomura-Takigawa et al. (2006)
Hepatitis C virus; NS3	Induces caspase-8	Prikhod’ko et al. (2004)
Influenza A; PB1-F2	Disrupts MMP	Chanturiya et al. (2004)
Human immunodeficiency virus type 1; Vpr	Rapid dissipation of the MMP and interaction with components of the PTPC	Jacotot et al. (2000)
SARS-CoV-1; gene 7a	Inhibit antiapoptotic Bcl-xL in cultured cells	Schaecher et al. (2007)
SARS-CoV-1; ORF-6	Induce caspase-3 mediated apoptosis	Ye et al. (2008)

## SARS-CoV-2 and Mitochondria

**Figure 2:**
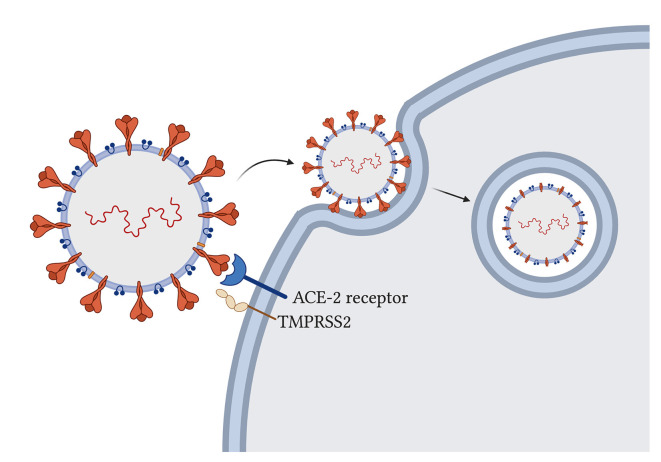
SARS-CoV-2 entry into host cells in the lung. Spike glycoproteins on the SARS-CoV-2 particle attach to human angiotensin-converting enzyme-2 for entry (Hoffmann et al., 2020). Host transmembrane serine protease 2 primes the spike protein for attachment (Hoffmann et al., 2020). Virus particle enters through endocytosis and spike proteins are cleaved (Ou et al., 2020).

The novel SARS-CoV-2 uses its spike glycoprotein on the angiotensin-converting enzyme-2 (ACE-2) host receptor (Cao et al., 2020) to enter human host cells and host transmembrane serine protease 2 (TMPRSS2) to prime the spike protein for attachment (Hoffmann et al., 2020; Figure 2). The virus particle enters the cell *via* endocytosis, and it has been proposed that the spike protein needs to be cleaved by host enzymes for viral entry to take place (Ou et al., 2020). ACE-2 influences mitochondrial functions and a lack of ACE-2 correlates with decreased ATP production and altered activation of NADPH oxidase 4 in the mitochondria, which is normally used for ROS production (Singh et al., 2020) that can both protect the cell by destroying pathogens or trigger the infected cell to go into apoptosis. With the SARS-CoV-2 virus using ACE-2 receptors for its entry, the availability of ACE-2 for its usual functions may be impaired and contribute to symptom development. Additionally, some studies have suggested that the TMPRSS2 from SARS-CoV-2 also influences mitochondrial function by acting on the estrogen-related receptor alpha, which is a nuclear receptor that regulates transcription of mitochondrial functions and energy homeostasis (Xu et al., 2018; Hoffmann et al., 2020; Singh et al., 2020).

Once inside the cell, SARS-CoV-2 triggers a massive inflammatory response. Through the innate immunity functions triggered upon viral infection detailed above, cytokines such as TNF-α, INF-γ, and interleukin-10 arrive at infected cells and cause an increase in mitochondrial ROS production through gene expression upregulation and electron transport chain modulation (Saleh et al., 2020). Mitochondrial ROS then stimulates additional proinflammatory cytokine production (Li et al., 2013) as the virus continues to persist, eventually leading to a “cytokine storm” in which over-inflammation can cause fatal harm if adaptive immunity does not take over in time. The immune response also causes the mitochondria to divert some energy away from ATP production to contribute to ROS production, which can harm the mitochondria in overwhelming amounts, leading to membrane permeabilization and apoptosis (Saleh et al., 2020). If severely damaged mitochondria release their contents into the cytosolic space, they stimulate the production of more cytokines such as IL-1β and IL-6 which are hallmarks for COVID-19 (Saleh et al., 2020).

Another mechanism of mitochondrial disruption employed by SARS-CoV-2 involves ferritin as evidenced by the high levels of ferritin in those with severe outcomes (Aguirre and Culotta, 2012). A normally functioning mitochondrion uses this iron to make heme, create iron-sulfur clusters, and store as mitochondrial ferritin (Saleh et al., 2020), but an overload of iron can lead to oxidative stress and impair mitochondrial function by reducing oxygen consumption by the mitochondria (Tang et al., 2020). Additionally, the ferritin overload can disrupt glucose tolerance in these cells with mitochondrial oxidative stress (Tang et al., 2020), which has implications for diabetic patients.

**Figure 3:**
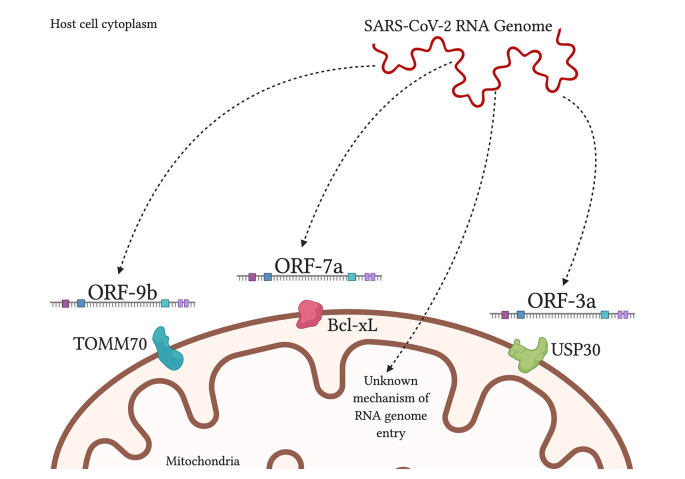
SARS-CoV-2 products localize to the mitochondria inside human host cells. SARS-CoV-2 RNA genome has been shown to localize in the mitochondrial matrix through an unknown mechanism. Viral protein ORF-9b interacts with translocase of outer mitochondrial membrane-70 (TOMM70), a host receptor that may affect activation of the interferon response (Gordon et al., 2020). Viral ORF-7a localizes to transmembrane protein Bcl-xL on the OMM, causing the promotion of apoptosis (Schaecher et al., 2007). Viral ORF-3a is theorized to localize to ubiquitin-specific protease-30 (USP30), which is typically involved in mitochondrial fission/fusion and mitophagy; and the sequence of ORF-3a that interacts with USP30 has been found in SARS-CoV-2 (Singh et al., 2020).

It is theorized that SARS-CoV-2 uses double-membrane vesicles derived from mitochondrial membranes to hide and protect itself inside the cell (Singh et al., 2020). This theory is based on evidence of HIV using ER-derived double-membrane vesicles (Somasundaran et al., 1994) and an observation that a point mutation in the coronavirus in rodents was shown to decrease ER-derived vesicles and increase localization of the virus to mitochondria at the same time (Clementz et al., 2008). Furthermore, a study found 5′ and 3′ untranslated regions on SARS-CoV-2 unique for mitochondrial localization, although further work needs to be done on this finding (Wu et al., 2020). When comparing SARS-CoV-1 and SARS-CoV-2, both are found to contain open reading frame ORF-9b, ORF-7a, and ORF-8b, which localize to the mitochondria, in the case of SARS-CoV-1, to alter MAVS function and mitochondrial function (Chen et al., 2007; Shi et al., 2014). SARS-CoV-2 additionally had ORF-3a present but ORF-3b absent (Singh et al., 2020; Figure 3). By encouraging the formation of double-membrane vesicles from the mitochondrial membrane or even the ER membrane, SARS-CoV-2 can safely avoid attacks from ROS and host proteases that threaten its survival. Meanwhile, the ROS is lingering around and can attack healthy tissue.

There is evidence of the SARS-CoV-1 ORF-9b causing mitochondrial fusion by the degradation of Drp1 by proteasomes (Shi et al., 2014; Holder and Reddy, 2020), and given the similarities in the genome, it is likely that the SARS-CoV-2 ORF-9b is lowering the amount of Drp1 as well, leading to more fusion. Mitochondrial fusion, which partly occurs *via* mitofusin 2, may lead to a hindered interferon response *via* inhibition of MAVS (Yasukawa et al., 2009). While this suggests that the lowered interferon numbers may take away from interferon-induced apoptosis specifically (Chawla-Sarkar et al., 2003), we must consider that SARS-CoV-1 is known to induce apoptosis *via* other factors such as ORF-6 and -7a (Schaecher et al., 2007; Ye et al., 2008). Comparing both SARS viruses indicates that SARS-CoV-2 may induce apoptosis when its need for the human host cell is over. Additionally, there is some evidence for ferroptosis or ferritin-induced apoptosis with iron overload. Defective mitochondria cannot metabolize iron as they normally would, leading to iron buildup and ferroptosis (Saleh et al., 2020). This all implies a greater number of cell death with COVID-19.

SARS-CoV-2 may also interfere with platelet count and coagulation, specifically with increasing coagulability and decreasing platelet count as the severity of COVID-19 increases (Tang et al., 2020; Terpos et al., 2020). Apart from the increasing risk of stroke, the increased coagulation and decreased platelets are impairing the cell’s ability to undergo mitophagy (Lee et al., 2016). When platelets cannot undergo mitophagy, they undergo apoptosis, which leads to increased thrombus formation; this is especially true in diabetic patients who suffer from oxidative-stress destroying their mitochondria yet hindering mitophagy (Lee et al., 2016). COVID-19 patients suffer from hyper inflammation and iron buildup, both of which are stressful to platelets, and thus contribute to the decreased platelet count (Saleh et al., 2020).

Men have had more severe outcomes with COVID-19 than women. While the cause is unknown, it has been speculated that the TMPRSS2 receptor is involved (Singh et al., 2020). TMPRSS2 can be induced by androgen, but not estrogen, and localize to the mitochondria to regulate mitochondrial function (Singh et al., 2020). Older individuals have also had worse outcomes. Aging is accompanied by a decrease in mitochondrial function, which has been shown to worsen the severity of viral illness and is also linked to numerous age-related diseases.

## Mitochondria and Aging

### Inflammation

“Inflammaging” is a phenomenon that describes worsened susceptibility to hyperinflammation among those who age (Hager et al., 1994; Soysal et al., 2016). Mitochondria may contribute to inflammaging when they release their intramitochondrial proteins and mitochondrial DNA (mtDNA) into cytosolic space upon membrane permeabilization, leading to recognition by intracellular immune receptors such as toll-like receptor-9 that activate neutrophil recruitment and cytokine production from monocytes (Jang et al., 2018). Moreover, levels of circulating mtDNA were found to steadily increase in individuals after 50 years of age (Pinti et al., 2014).

Inflammasomes, or multiprotein complexes that are a part of innate immunity signaling, are found to be involved in aging and particularly in age-related diseases through their ability to activate caspase-1 (Furman et al., 2017). Caspase-1 activation can be harmful to mitochondria, and MAVS, mitochondrial membrane cardiolipin, ROS, and mtDNA from damaged mitochondria were all found to activate inflammasomes (Jang et al., 2018). Furthermore, SARS-CoV-2 may activate inflammasomes (Shah, 2020), further putting the elderly at risk of hyperinflammation.

### mtDNA Mutations and Increased ROS

Mitochondrial DNA sees more mutations than nuclear DNA, and age-related increases in mutated mtDNA and increased ROS levels have been causally connected (Reddy and Beal, 2005; Reddy, 2006; Kuka et al., 2013; Kang et al., 2016; Oliver D. M. A. and Reddy, 2019). This may be due to the findings that mtDNA is placed spatially close to the ROS-producing machinery of the respiratory chain (Chistiakov et al., 2014) and mutations in mtDNA can lead to increased ROS production and mitochondrial malfunction (Wallace, 2010). While the most widely accepted theories suggest that ROS only led to detrimental effects on mitochondrial health, there has also been evidence that some ROS is required to balance redox reactions and stimulate anti-oxidant functions to keep the cell alive—even contributing to longevity (Schulz et al., 2007; Yang and Hekimi, 2010). Nevertheless, ROS in amounts larger than necessary cause age-related cellular damage (Chistiakov et al., 2014). Seeing as SARS-CoV-2 invokes ROS production indirectly, an aged person’s cells may face an even greater amount of ROS exposure upon infection with this virus compared to healthy young individuals.

### Quality Control of Mitochondria

In combination with lessened ATP production, there is a decrease in mitophagy as a person ages (García-Prat et al., 2016), which not only contributes to unregulated inflammasome activity (Jang et al., 2018) but also an accumulation of mitochondria that may no longer produce energy efficiently. Mitophagy is a protective function of the cell that keeps inflammation at a manageable level by removing damaged mitochondria that could contribute to hyper inflammation, especially among already susceptible older patients. Without autophagy or mitophagy, levels of ROS rise and cause oxidative stress and related tissue damage (Yan and Finkel, 2017).

**Figure 4:**
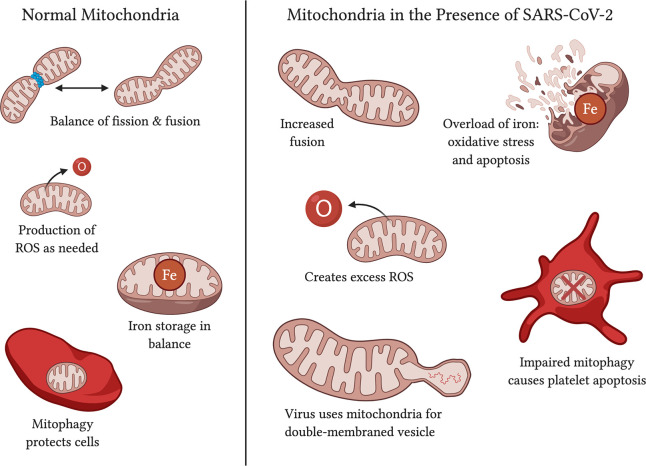
Functions of normal mitochondria vs. effects on mitochondria in the presence of SARS-CoV-2. Normal mitochondria have a balance on fission and fusion, produce ROS only in quantities necessary, have balanced iron storage, and use mitophagy to upkeep functional mitochondria and protect the cell against damage. In the presence of SARS-CoV-2, mitochondria may have increased fusion (Shi et al., 2014; Zhang et al., 2020), an excess of ROS production (Kuka et al., 2013; Kang et al., 2016), too much iron in storage (Saleh et al., 2020), and impaired mitophagy that leads to platelet apoptosis (Lee et al., 2016; Tang et al., 2020). SARS-CoV-2 affects mitochondria may also serve as a source of double-membraned vesicles for the virus to travel in (Singh et al., 2020).

Mitochondrial fission and fusion are important functions that change with age and in neurodegenerative diseases (Reddy et al., 2011; Kandimalla and Reddy, 2016; Oliver D. and Reddy, 2019). Mitochondrial biogenesis occurs from growth to increase mass and division to increase the number (Chistiakov et al., 2014; Pradeepkiran and Reddy, 2020). Despite reduced mitophagy and dysfunctional mitochondria, the overall mitochondrial count decreases with age in skeletal muscle (Crane et al., 2010) and this may be due to decreased biogenesis (Chistiakov et al., 2014). The fission and fusion balance tend to fall off with age, with fission decreasing and leading to poorer quality control for the mitochondria as well as decreased mitophagy (Chistiakov et al., 2014). This may be due in part to dysregulation of proteins that are involved in fission, including DRP1 (Udagawa et al., 2014). While fusion has been found to protect mitochondria against starvation-induced autophagosomal degradation (Shi et al., 2014), an unbalanced ratio may be harmful. As the ratio for fusion increases, so does the difficulty for a cell to dispose of damaged, overlarge mitochondria. Improperly structured mitochondria become stressed and turn to increased ROS production (Chistiakov et al., 2014), adding to the initial problem (Figure 4). SARS-CoV-1 is observed to stimulate mitochondrial fusion (Shi et al., 2014), and SARS-CoV-2 can be expected to do the same given the genomic similarities. As an elderly person is already subject to increased mitochondrial fusion, they are in a worse position to fend off the additional burden of a virus with such capability. However, some studies indicate an increase in fission in elderly animal models (Chaudhari and Kipreos, 2017), so the evidence behind an increase in one function over the other is not complete and still requires further study and elaboration.

The impaired mitochondria quality through defective mitophagy and fusion/fission imbalance may contribute to a decrease in energy production with increasing age. A study on skeletal muscle found that mitochondrial respiration capacity declined by about 50% in older patients compared to young patients, with an accompanying decline in ATP (Conley et al., 2000). Aging was also shown to decrease ATP synthase activity (Frenzel et al., 2010). As fatigue and muscle weakness is among the symptoms of COVID-19, those with aged mitochondria are matched to this shortness of energy.

### Oxidative Stress and Calcium Dyshomeostasis in Mitochondria

Another important factor to consider in the mitochondria’s role in aging has to do with mitochondrial permeability transition pores (mPTP), which sit on the IMM and open up in response to excessive calcium in the mitochondria (Panel et al., 2018). The mPTP’s sensitivity to calcium is increased when the cell is under oxidative stress (Halestrap and Richardson, 2015). As humans age, basal calcium levels increase and affect mPTPs to open their pores more often (Panel et al., 2018). This is observed to have a more detrimental effect on cardiac muscle because calcium is used as a communication tool between the sarcoplasmic reticulum and mitochondria. Without the control of mPTPs in releasing calcium into the cytosol in regulated amounts, the failure of calcium transfer, decreased energy production, and increase in oxidative stress may altogether contribute to heart failure in older individuals (Szalai et al., 2000; Kohlhaas and Maack, 2013; Fernandez-Sanz et al., 2014). Myocardial infarctions, a type of heart attack, are also linked to mPTP activation; studies found that mPTP opening and apoptosis are increased in aged cardiac cells (Fernandez-Sanz et al., 2015), possibly as the result of oxidative stress in aging (Ferrara et al., 2008).

### Neurological Diseases

The role of mutated mtDNA, oxidative damage, decreased energy production and increased ROS production all come together in age-related neurological diseases. Alzheimer’s disease is associated with increased free radical production and oxidative stress, mitochondrial dysfunction, and impaired ATP production (Beal, 2005; Reddy and Beal, 2005, 2008). While definitive evidence for an increase in mtDNA changes has not yet surfaced, a few studies point in that direction (Coskun et al., 2004) as well as towards disrupted axonal transport of mitochondria in AD neurons (Stokin et al., 2005; Calkins et al., 2011). Alzheimer’s causes a buildup of amyloid precursor protein and amyloid-beta, both of which are found on the mitochondrial membrane (Crouch et al., 2005; Manczak et al., 2004), where they induce increased free radical production, decrease cytochrome oxidase activity, and decrease ATP production (Parker et al., 1990; Smith et al., 1996; Gibson et al., 1998).

Parkinson’s disease analysis shows disease-specific proteins in mitochondrial membranes and matrix (Reddy, 2009; Reddy and Reddy, 2011). Additionally, PINK1 protein overexpression disrupts the MMP which leads to impaired respiration (Reddy, 2009; Pradeepkiran and Reddy, 2020). Parkin is another protein, a ligase, that is associated with the OMM and induces free radical production (Reddy, 2009). Huntington’s disease shows a mutated huntingtin protein bound to the OMM and also induces free radical production, and this disease shows the dysfunctional movement of mitochondria in cells affected by Huntington’s (Reddy, 2009; Reddy et al., 2009; Reddy and Shirendeb, 2012). Normally functioning SOD1 proteins seek out and counteract ROS to protect cellular function, but in Amyotrophic Lateral Sclerosis (ALS), mutated SOD1 contributes to oxidative stress built up in free radicals and mitochondrial dysfunction (Reddy, 2009; Reddy and Reddy, 2011).

Given the evidence for mitochondrial involvement in both age-related diseases and SARS-CoV-2, it is not difficult to see how it is easier for the virus to induce more severe outcomes in those with compromised mitochondria, especially those with neurological diseases and diabetes.

### SARS-CoV-2 and Aging

Older individuals lose acquired immunity as they age, and the innate immune system tries to compensate for that by increasing inflammation signals such as CRP-1, IL-6, and fibrinogen among others (Franceschi et al., 2007; Soysal et al., 2016). Notably, C-reactive protein and IL-6 are significantly increased in the response to SARS-CoV-2 in severely ill patients (Gong et al., 2020). Fibrinogen, which is involved in the coagulation process that contributes to thrombosis formation and vascular weakening, is also observed to be increased in response to SARS-CoV-2 (Tang et al., 2020; Terpos et al., 2020). Sustained inflammation can lead to cell destruction and apoptosis. SARS-CoV-2 can cause hyper inflammation, and in an elderly person prone to an over-stimulated inflammatory response, this combination can make them more susceptible to death by “cytokine storm” and offers a possible explanation for the increased mortality among the elderly population (Jeyaraman et al., 2020).

Aging cells embody senescence in part from an increase in mitochondrial dysfunction (Wiley et al., 2016). Given the negative effects on mitochondrial health by SARS-CoV-2 discussed previously, an aged person is starting with already weakened mitochondria and facing a disease that affects mitochondria. This progression can only lead to worsened outcomes. Senescence also affects macrophages, which have protective effects on the lungs during a SARS-CoV-2 infection; without properly functioning macrophages, the body’s response to SARS-CoV-2 will be weaker (Liu et al., 2020). Older individuals were also found to have increased levels of mtDNA in the cytoplasm (Pinti et al., 2014), and due to mtDNA’s role in inducing innate immunity and increasing inflammation, this is likely another mechanism that contributes to the lethal levels of inflammation seen in older COVID-19 patients (Singh et al., 2020).

### Diabetes and Obesity in SARS-CoV-2

ACE-2 is an enzyme that serves in the renin-angiotensin system to adjust water volume as needed and holds the receptor for SARS-CoV-2 to enter in the lungs. ACE-2 works as a “negative” regulator by cleaving angiotensin II so that it does not overwhelm the body with increased blood pressure (Obukhov et al., 2020). Increasing age and uncontrolled diabetes both correlate with a decreasing amount of ACE-2 expression (Xie et al., 2006; Obukhov et al., 2020). The use of ACE-2 receptors for cell entry by SARS-CoV-2 can exacerbate the lower availability of the enzyme for its anti-inflammatory purposes and contribute to unchecked blood pressure and inflammation in diabetics.

Diabetics who control their condition with ACE-inhibitors and angiotensin II-blockers see some upregulation of ACE-2, although not likely to above-normal levels (AlGhatrif et al., 2020) but this could protect against severe COVID-19. Those who take the drug Metformin, usually for diabetes, have also seen less severe COVID-19 infections compared to those who did not (Scheen, 2020).

Those with obesity have increased adipose tissue which comes with associated meta-inflammation, a state of chronic, low-grade inflammation (Mauvais-Jarvis, 2020). This creates an environment that makes it more likely for SARS-CoV-2 to trigger a cytokine storm and cause the more severe, and sometimes lethal, consequences of COVID-19 (Mauvais-Jarvis, 2020). With the slightly lowered anti-inflammatory capabilities in diabetics *via* loss of ACE-2 receptors, the meta-inflammation of obese patients, and the lowered quality of mitochondrial function and protection in elderly patients, it can be theorized that older patients with comorbidities have a combination of risky features that make SARS-CoV-2 infection more likely to be severe.

## Treatments and Prevention for SARS-CoV-2

Medications currently approved for use in COVID-19 patients include repurposed drugs such as hydroxychloroquine, ribavirin, lopinavir-ritonavir, darunavir, and cobicistat, favipiravir, arbidol, remdesivir, and combination therapies (Bhatti et al., 2020; Kandimalla et al., 2020), most of which are antivirals against other viruses. Vaccines against SARS-CoV-2 are still in their infancy and are variously targeting spike proteins (Kandimalla et al., 2020), but they may not be as effective in elderly patients due to the decline of adaptive immunity with age.

To target mitophagy, patients could use calorie restriction to conserve existing mitochondrial shape (Khraiwesh et al., 2014), or polyamine spermidine to increase autophagy/mitophagy (Eisenberg et al., 2016). Urolithin A, found in pomegranates, is also known to encourage mitophagy (Ryu et al., 2016). By improving mitophagy, we reduce inflammation and give the elderly a better chance of surviving the immune response. Experimentally, mitochondrial transfer from bone marrow stromal cells is effective in protecting against acute lung injury (Islam et al., 2012), and is a technique that can be used here, although there is not much evidence of current use.

Exercise has been shown to not only protect against mitochondrial decline but aging itself (Fiuza-Luces et al., 2013; Garatachea et al., 2015). Maintaining muscle mass and strong vasculature encourages the body to keep your mitochondria alive and well. Over years of exercise, your body adapts to become more stress-resistant, homeostatic, and protected against chronic illnesses and cancers (Nilsson et al., 2019). When it comes to the lungs, breathing exercises have been recommended to be useful in training your respiratory muscles and increase lung capacity; while there are yet to be any controlled experiments on the effectiveness of this method, physical therapists and physicians around the globe are recommending the preventative measure [Lien, 2020; American Lung Association (ALA), 2020].

**Figure 5:**
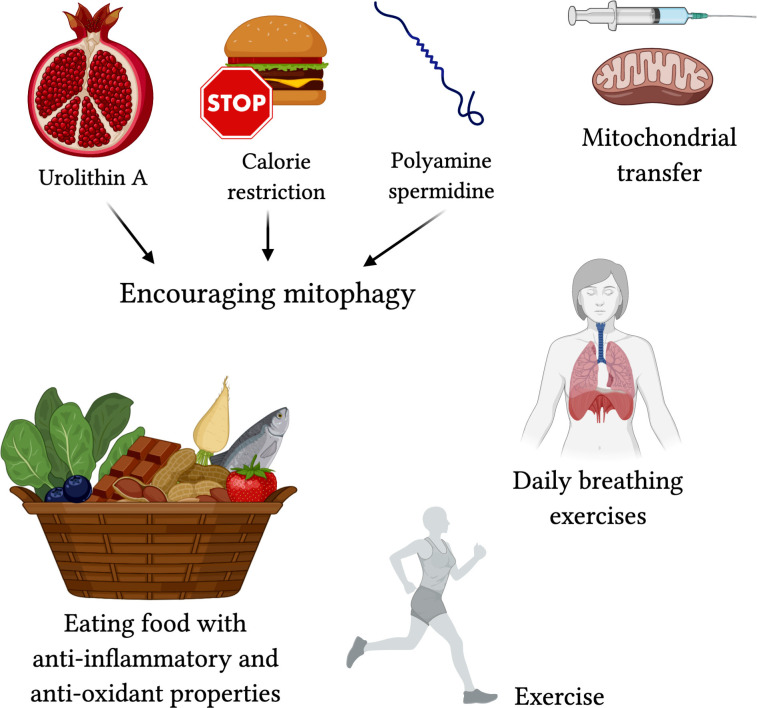
Proposed treatments for targeting mitochondrial-centered dysfunction in COVID-19. Encouraging mitophagy with Urolithin A (found in pomegranate juice; Ryu et al., 2016), calorie restriction (Khraiwesh et al., 2014), and polyamine spermidine (Eisenberg et al., 2016) may reduce severe effects of COVID-19. Mitochondrial transfer (Islam et al., 2012) may protect against acute lung injury. Exercise, breathing exercises, and foods high in anti-oxidants and with anti-inflammatory properties are lifestyle changes that can contribute to better protection against the severe respiratory symptoms in COVID-19.

Along those lines, consuming foods with high antioxidant properties, such as raw cacao, berries, matcha, pecans, artichokes, beets, kale, and spinach can help prevent the damage caused by ROS. Anti-inflammatory foods such as heart-healthy oils, fish, fruits, nuts, garlic, herbs, and chocolate do not go amiss either (Figure 5).

## Conclusions and Future Directions

There are many avenues involving mitochondria and their roles in inflammation that can offer answers as to why SARS-CoV-2 is impacting the elderly population so harshly, especially those that have comorbidities. Therefore, the role of mitochondria should not be ignored in the direction that treatment discovery takes. There are several links between aging mitochondria and weakened immunity; the avenues include over-stimulated or sustained inflammatory responses with interferon and cytokine release, regulation of fission and fusion, mitochondrial biogenesis, and interference of apoptosis and mitophagy. Many pathogens have shown a tendency to affect mitochondria as a way to influence host behavior once inside a cell by affecting these functions, from bacteria to parasites to viruses similar to the SARS-CoV-2.

SARS-CoV-2 enters the cell *via* the ACE-2 receptor and sends its genetic material towards the mitochondria to influence ROS production, mitophagy, iron storage, platelet coagulability, and cytokine production stimulation. These functions are already suffering in aging patients. In those with comorbidities, the impaired mitochondrial functions amplify other issues that contribute to severe outcomes, such as ferritin storage in diabetes and increased coagulability in heart disease. This could provide a reason as to why older, comorbid patients have the most severe outcomes with COVID-19 and offer one direction for developing drug therapy.

While scientists around the world grapple with finding the definitive cure to the novel disease that is COVID-19, there are many things one can do at home to give themselves the best chance at survival. Apart from medications targeted at strengthening the mitochondria, exercise, fresh foods, breathing practices, and general preventative medicine practices can help the body protect itself.

## Author Contributions

PR contributed to the conceptualization and formatting of the article. RG and PR are responsible for writing, original draft preparation, and finalization of the manuscript. PR is responsible for funding acquisition. All authors contributed to the article and approved the submitted version.

## Conflict of Interest

The authors declare that the research was conducted in the absence of any commercial or financial relationships that could be construed as a potential conflict of interest.
